# The impact of access to immunization information on vaccine acceptance in three countries

**DOI:** 10.1371/journal.pone.0180759

**Published:** 2017-08-03

**Authors:** Lori K. Handy, Stefania Maroudi, Maura Powell, Bakanuki Nfila, Charlotte Moser, Ingrid Japa, Ndibo Monyatsi, Elena Tzortzi, Ismini Kouzeli, Anthony Luberti, Maria Theodoridou, Paul Offit, Andrew Steenhoff, Judy A. Shea, Kristen A. Feemster

**Affiliations:** 1 Department of Pediatrics, The Sidney Kimmel Medical Center at Thomas Jefferson University, Philadelphia, Pennsylvania, United States of America; 2 Division of Infectious Diseases, Nemours/Alfred I. duPont Hospital for Children, Wilmington, Delaware, United States of America; 3 Vaccine Education Center, Children’s Hospital of Philadelphia, Philadelphia, Pennsylvania, United States of America; 4 Collaborative Center for Clinical Epidemiology and Outcomes Research, Athens, Greece; 5 Global Health Center, Children’s Hospital of Philadelphia, Philadelphia, Pennsylvania, United States of America; 6 Botswana-UPenn Partnership, Gaborone, Botswana; 7 Division of Infectious Diseases, Children’s Hospital of Philadelphia, Philadelphia, Pennsylvania, United States of America; 8 Robert Reid Cabral Hospital, Santo Domingo, Dominican Republic; 9 Child Health Division, Ministry of Health, Gaborone, Botswana; 10 Department of Pediatrics, Perelman School of Medicine, University of Pennsylvania, Philadelphia, Pennsylvania, United States of America; 11 Botswana-UPenn Partnership & Department of Pediatric & Adolescent Health, University of Botswana, Gaborone, Botswana; 12 Department of Medicine, Perelman School of Medicine, University of Pennsylvania, Philadelphia, Pennsylvania, United States of America; University of Toronto, CANADA

## Abstract

**Introduction:**

Vaccine acceptance is a critical component of sustainable immunization programs, yet rates of vaccine hesitancy are rising. Increased access to misinformation through media and anti-vaccine advocacy is an important contributor to hesitancy in the United States and other high-income nations with robust immunization programs. Little is known about the content and effect of information sources on attitudes toward vaccination in settings with rapidly changing or unstable immunization programs.

**Objective:**

The objective of this study was to explore knowledge and attitudes regarding vaccines and vaccine-preventable diseases among caregivers and immunization providers in Botswana, the Dominican Republic, and Greece and examine how access to information impacts reported vaccine acceptance.

**Methods:**

We conducted 37 focus groups and 14 semi-structured interviews with 96 providers and 153 caregivers in Botswana, the Dominican Republic, and Greece. Focus groups were conducted in Setswana, English, Spanish, or Greek; digitally recorded; and transcribed. Transcripts were translated into English, coded in qualitative data analysis software (NVivo 10, QSR International, Melbourne, Australia), and analyzed for common themes.

**Results:**

Dominant themes in all three countries included identification of health care providers or medical literature as the primary source of vaccine information, yet participants reported insufficient communication about vaccines was available. Comments about level of trust in the health care system and government contrasted between sites, with the highest level of trust reported in Botswana but lower levels of trust in Greece.

**Conclusions:**

In Botswana, the Dominican Republic, and Greece, participants expressed reliance on health care providers for information and demonstrated a need for more communication about vaccines. Trust in the government and health care system influenced vaccine acceptance differently in each country, demonstrating the need for country-specific data that focus on vaccine acceptance to fully understand which drivers can be leveraged to improve implementation of immunization programs.

## Introduction

Vaccines are one of the most important public health achievements in history resulting in significant decreases in the prevalence of many childhood diseases [1]. Vaccination rates have steadily increased in both developed and developing countries through the World Health Organization’s Expanding Program on Immunization and various public-private partnerships. However, disparities in rates of new vaccine adoption and sustained vaccination rates across different regions persist. In addition, an increase in the number of communities in both developed and developing countries with low or decreasing vaccination rates has resulted in disease outbreaks [2,3]. While access to health care services can lead to disparities in vaccination rates, vaccine acceptance is another critical component of sustainable immunization programs. Studies have suggested that vaccine-hesitant individuals, who hold various degrees of indecision about specific vaccines or vaccination in general, can significantly contribute to decreases in immunization rates and slow uptake of newly introduced vaccines [4,5,6,7,8].

In light of these changing trends, there is increased focus on defining vaccine hesitancy and identifying key determinants that can be addressed by immunization programs, although there is not significant evidence supporting specific interventions to reduce hesitancy [9,10]. A recently published review suggests that region- or country-specific research is crucial given the complex array of individual, sociocultural, and political factors that influence vaccine acceptance [6]. In international studies, several factors have been identified that affect vaccine acceptance, including lack of knowledge about the need for vaccines, misperceptions about vaccines and vaccine-preventable diseases, fear of side effects after vaccination, inconsistent vaccine availability, lack of trust in the health system, and history of a negative interaction with immunization providers [11,12,13,14,15,16,17]. In a multinational study of immunization managers in low- and middle-income countries, a variety of views were presented, but few managers were concerned about vaccine hesitancy [18]. A study of health care workers in Greece, France, Croatia, and Romania demonstrated the predominance of concerns about vaccine safety [19]. In developed countries in Europe facing financial crisis, health inequalities are expected, with concern that vaccination coverage will decline [20]. Reliable sources of information about immunizations vary by country. In settings with expanding immunization programs, access to information about newly introduced vaccines may be particularly important. Globalization has resulted in a rapid increase in information exchange. The effect of pro- and anti-vaccine news from developed countries on worldwide attitudes toward vaccination may significantly impact attitudes toward vaccines [21,22].

We explored attitudes and beliefs related to vaccine acceptance and the methods of communication about vaccines leading to vaccine acceptance in three countries in diverse regions of the world and with immunization programs in various stages of development: Botswana, the Dominican Republic (DR), and Greece. Botswana and the DR are middle-income countries in different parts of the world with expanding immunization programs within a developing public health system, whereas Greece has an established health care delivery system that has been acutely affected by significant resource limitations [23]. Because current studies suggest that vaccine acceptance in different regions is dependent on factors specific to that sociopolitical and cultural environment, independent evaluation of these factors in different regions is essential [6]. Investigating attitudes toward childhood vaccines and the perceived influence of information sources in these settings can provide important insights regarding vaccine acceptability as new vaccines are introduced to ensure ongoing sustainability of immunization programs.

## Materials and methods

### Study settings

This qualitative study was conducted in two middle-income countries, Botswana and the DR, where immunization programs are primarily supported through the public health system, and Greece, an upper-income country with an immunization program supported through both public and private health care systems. These three countries were selected to identify both similarities and differences in vaccine acceptance in light of the varied cultures, sociopolitical environments, and stages of development of each nation’s vaccination program, as detailed in Table 1. Due to this complexity of factors, it is important to measure attitudes in countries that have different immunization practices. Our established relationships with these countries allowed us to carry out this research and prepare for next steps in increasing vaccine acceptance.

**Table 1:** Study sites.

	Botswana	Dominican Republic	Greece
**GDP (2014)** [24]	$7757	$6075	$21683
**National Immunization Program**	WHO EPI	WHO EPI (*Programa Ampliado de Inmunizacion*)	National Immunization Program (NIP)–Ministry of Public Health and Social Solidarity
**Vaccine Cost**	Free of charge through public sector	Free of charge through private sector	Free of charge through private sector
**Newly Introduced Vaccines** for children <5 years of age [25]	PCV (2012)	PCV (2009)	PCV (2006)
	Rotavirus (2012	Rotavirus (2012)	
**Private Sector Vaccination** [26]	Minimal	Minimal	65%–70%*
**Vaccination Rates, 2014** [27]	99% PCV1	94% PCV1	99% PCV1
	81% PCV3	27% PCV3	96% PCV3

GDP, gross domestic product; WHO EPI, World Health Organization Expanded Program on Immunization; PCV, Pneumococcal conjugate vaccine.

*Publically funded vaccines can be administered by either a private or public physician.

Botswana has a rapidly growing economy but is challenged by the third-highest rate of HIV/AIDS in the world [28]. The DR also has a growing economy but has significant income disparities. While national estimates for immunization rates are high in both Botswana and the DR, there is significant variation in coverage rates by district, suggesting that implementation of current vaccination recommendations is not uniform [29,30]. In each location, a small proportion of children receive care through the private sector where they may also receive vaccines, particularly those that are not included in the national vaccine program. Both settings have a large immigrant population—from Zimbabwe in Botswana and from Haiti in the DR—and these children are eligible to receive immunizations through each country’s National Immunization Program (NIP).

In Greece, the NIP includes universally recommended vaccines free of charge to all resident children, including immigrants [23]. While children can receive vaccines in primary health centers and non-governmental organizations at no additional cost, the majority receive vaccines through the private sector [26]. An economic crisis has engulfed Greece since 2008, which has undoubtedly been detrimental to the health care system [31]. For example, many families lost public health insurance coverage because of unemployment, creating significant gaps in insurance benefits. Although research at the beginning of the crisis demonstrated that incomplete vaccination was not associated with insurance status but rather was driven by socioeconomic factors such as parent age and occupation, this relationship is incompletely understood [23].

### Study design

We implemented focus groups among caregivers of children younger than 5 years and immunization providers to identify salient themes associated with vaccine acceptance and hesitancy, to investigate knowledge regarding childhood vaccines, and to explore content of and access to information about vaccines and vaccine-preventable diseases. Qualitative methods with focus groups were utilized to encourage candid responses, to allow participants to build on each other’s ideas, and to allow participants to focus the discussion on topics most relevant to their experiences [32]. For caregivers, we included any individual who was the parent or guardian of a child 5 years of age or younger who made decisions regarding vaccination for that child, regardless of the caregiver’s attitudes toward vaccination. For immunization providers, we included any clinician (public health practitioners, nurses, or physicians) who administered vaccinations to children 5 years of age or younger. Participants were compensated for their time with refreshments and reimbursement of transportation costs at all sites. In Botswana and the DR, caregivers and providers were recruited from clinics in low-, middle-, and high-income neighborhoods across urban, peri-urban, and rural communities with a range of sociodemographic characteristics and immunization rates to capture a comprehensive array of attitudes toward vaccination. In Greece, caregivers were recruited from kindergarten networks in communities of low-, middle-, and high-income neighborhoods, while providers were recruited through public and private hospitals and government agencies. All focus group participants signed an informed consent document, with review and approval by the Institutional Review Boards of the University of Pennsylvania and Agia Sofia Children’s Hospital in Greece, Conabios in the DR, and the Botswana Ministry of Health.

### Focus groups

**Fig 1:**
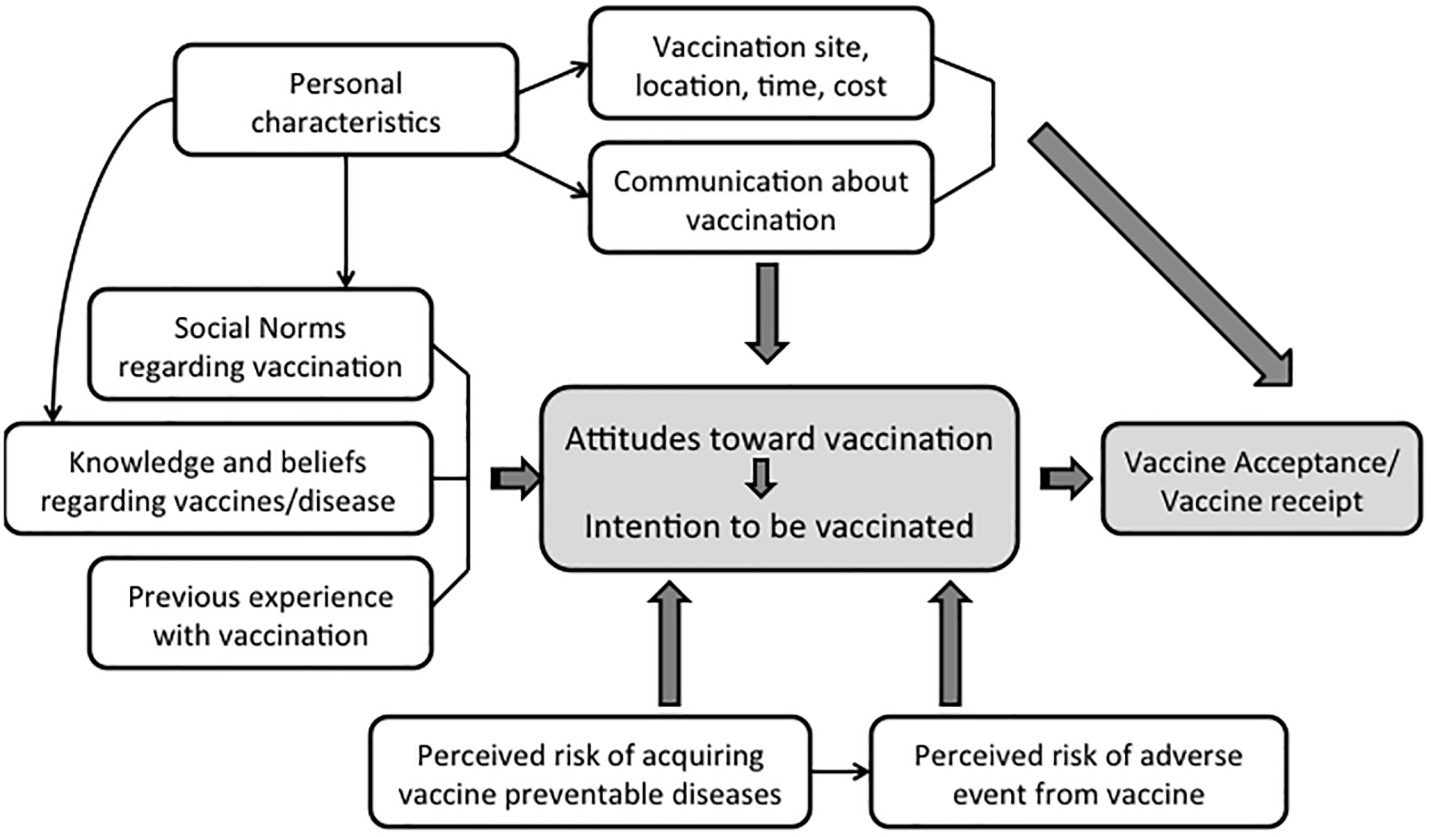
Conceptual model demonstrating the interrelationship of factors that drives attitudes toward vaccination.

The focus group guide was developed based upon a conceptual framework grounded in the health belief model but also integrating theoretical models related to vaccine hesitancy [33,34] (Fig 1). The conceptual framework and focus group guide were developed in consultation with a study team member with expertise in qualitative research methods (JS). Themes explored included three domains: (1) knowledge, communication, and the health care system; (2) attitudes and beliefs; and (3) logistics of vaccine delivery. Questions for both the focus groups and interviews were read from a standardized guide eliciting knowledge and beliefs about vaccines and vaccine-preventable diseases, sources of vaccine-related information, influence of popular media on vaccine-related beliefs, facilitators and barriers to obtaining vaccines, and attitudes toward the health care system. All focus group moderators and interviewers were trained by a qualitative researcher (JS). The focus group and interview guides were translated and back-translated into Setswana, Greek, and Spanish.

Focus groups led by trained research associates were conducted in Setswana, English, Greek, or Spanish, depending on the preference of the participants. Focus groups for providers and caregivers were conducted independently. Those groups with one or two participants were conducted as semi-structured interviews. Sessions lasted approximately 90 minutes and were audiotaped. Sessions were then transcribed and translated into English. Facilitators of focus groups fluent in native languages validated translation quality.

### Analysis

Translations uploaded into NVivo 10 (QSR International, Melbourne, Australia), a qualitative data analysis software program, were analyzed for common themes through a deductive approach based on the conceptual framework. As new themes emerged, an inductive approach was used to add to the coding schema. Two independent coders coded the transcripts, with discrepancies rectified on the basis of review in an iterative process. Kappa values were calculated between the initial two coders after reviewing and modifying discrepancies. If there was lack of agreement between the two coders after review, a third coder evaluated the discordance to reach a Kappa value of 1.

The coding schema on completion was reorganized from three domains into four domains: (1) knowledge and communication, (2) interactions with the health care system; (3) attitudes and beliefs; and (4) logistics of vaccine delivery. Within each theme, comments were categorized into nodes within NVivo that were neutral toward, supported, or presented a barrier to vaccine acceptance.

## Results

We conducted 37 focus groups and 14 semi-structured interviews among 96 providers and 153 caregivers across three countries (Table 2). The majority of participants received or provided care within a public sector setting. Male and female providers in different practice settings in all involved communities were recruited.

**Table 2:** Demographics of focus group participants.

Community (Country)	Practice Setting	Practice Type	Number of Providers	Number of Caregivers
Gaborone (BW)	Urban	Public	6	12^§^
Gaborone (BW)	Urban	Private	6	N/A
Kgatleng (BW)	Peri-urban	Public	5	3
Lobatse (BW)	Rural	Public	12	4
Ramotswa (BW)	Peri-urban	Public	4	3
Santo Domingo (DR)	Urban	Private	5	8
San Pedro de Macoris (DR)	Peri-Urban	Public	5	7
Santo Domingo (DR)	Urban	Public	23	12
Consuelo (DR)	Rural	Public	4	32
Kifissia (GR)	Urban	Mixed	8	7
Papagou-Xolargos (GR)	Urban	Mixed	5	9
Downtown Athens (GR)	Urban	Mixed	6	9
Egaleo-Haidari (GR)	Urban	Mixed	-	4
Keratea (GR)	Peri-urban	Mixed	-	13
Elefsina (GR)	Peri-urban	Mixed	-	7
Nafplio (GR)	Peri-urban	Mixed	7	-

BW, Botswana; DR, Dominican Republic; GR, Greece.

^**§**^ Public and private caregivers were not differentiated.

Within the four domains, there were 23 themes, although two were specific to Greece. In the Botswana focus groups, 945 comments were included in the final analysis with 1122 attributions to nodes (~20% of comments were attributed to >1 node). Ninety-one percent of Kappa values using NVivo software were above 0.6, with a mean of 0.9. In the DR focus groups, 1076 comments were included in the final analysis with 1236 attributions to nodes (~15% of the comments were attributed to >1 node). Ninety-eight percent of Kappa values using NVivo software for the primary coders were above 0.6 with a mean of 0.97. In the Greece focus groups, 1080 comments were included with 1225 attributions to nodes (~14% of the comments were attributed to >1 node.) Ninety-seven percent of Kappa values using NVivo software for the primary coders were above 0.6 with a mean value of 0.97. The frequency with which each theme was discussed, either as supporting vaccine acceptance, creating a barrier to vaccine acceptance, or without impact on vaccine acceptance, was tallied to identify prevalent themes (S1 Appendix). The numbers next to each node are the total number of comments elicited across all groups. As study methodology allowed participants to speak freely about topics, these values should not be considered precise measurements of importance. The domains of logistics of vaccine delivery and attitudes and beliefs confirmed published literature, while the domains of knowledge and communication, as well as interactions with the health care system, are highlighted in this manuscript.

### Domain: Knowledge and communication

In recognizing that knowledge about vaccine-preventable diseases, vaccines, vaccine efficacy, and vaccine safety influences vaccine acceptance, it is critical to assess how caregivers and providers build their knowledge base. Results demonstrate the relative impact of information from health care providers, the media, community members, and society in each country. A dominant theme that emerged was the recognition of health care providers as the main source of vaccine information for caregivers with indications that they relied on them more than other information sources. Similarly, health care providers recognized their role as information sources for their patients and looked to medical literature primarily as an information source to maintain their knowledge. Representative quotes are highlighted in Table 3.

**Table 3:** Theme of health care providers and medical literature as information source.

	**Caregivers’ Comments**
Botswana	• “Actually, we are taught all that information at the clinics. They even show us how to check what dates the child has to be brought back for subsequent doses.” • “I have never had any stories but if I come across something that gets me confused I would go to the clinic.” • “If I come across something [in the media] that gets me confused, I would go to the clinic.” • “The Clinic is the most reliable source.”
DR	• “I have also acquired information in the medical centers, when I have taken my child to get vaccinated.” • “The Ministry of Public Health has implemented some televised programs, they even have a website. They use mass media that the public has access to, mostly to encourage them.” • “I don’t know why you wouldn’t get vaccinated because the card tells you the appointment date when you have to go and get it.”
Greece	• “If you want to and you have the time and willingness there is information.” • “And the pediatrician is the one that will encourage parents to do or not to do vaccines, and parents usually rely on pediatricians.”
	**Health Care Providers’ Comments**
Botswana	• “Normally when they introduce new vaccines, there is some kind of workshop or seminar we engage in just to learn about that vaccine, the importance of it. But I believe that even at our institutions we learned, at our institutions where we were doing the nursing course, we learned about these conditions, measles and everything, and the importance of vaccines there as well.” • “We are confident because every time before the vaccine is introduced in a health facility we go for a workshop, we get equipped.”
DR	• “Actually, we are usually being asked to attend conferences or workshops. Internet, books and other resources. And conferences every so often. There are conferences exclusively on immunization and they request that everybody attends, and we participate in that.” • “The majority [of parents] respond positively to recommendations that pediatricians make [clearly], about vaccines.” • “At conferences, we also have a vaccine society in our country that manages continuing medical education.”
Greece	• “I have been called as a pediatrician to talk to our primary school about vaccines.” • “Yes, but it is pediatrician’s responsibility to inform.”

DR, Dominican Republic

Within this theme in Botswana and the DR, information from health care providers was described as positively supporting vaccine acceptance, while in Greece, many participants felt that they received information from health care providers that negatively affected vaccine acceptance. Similar trends were noted for both community members and media as information sources. These themes were mentioned less frequently across all sites, but participants from Greece were more likely to consider messages from community members or popular media as negatively affecting vaccine acceptance.

Forms of media used by study participants varied between countries, with emphasis on radio, television, and newspapers in Botswana; television and Internet in the DR; and the internet in Greece. Representative quotes from caregivers and providers are highlighted in Table 4. In Botswana, participants noted that media delivered positive messages about vaccines usually in the form of public service announcements or general updates on new vaccines that became available. If media messages were negative or raised questions, participants indicated that they would confirm the information with health care providers rather than trust the media’s negative message about vaccines. In the DR, while the majority of information received through media was reported as supporting acceptance, many participants pointed out that access to television or the internet is not consistent throughout the country, possibly limiting the impact of this information. In Greece, caregivers reported pediatricians as their main source of information for vaccination. However, caregivers were frequently exposed to media messages, especially through the internet. A significant number of caregivers expressed a desire to discuss any negative concerns with their family doctors before making vaccine decisions. However, some caregivers also described a direct influence of media messages on their decision-making regarding vaccination, regardless of consultation from their family doctor.

**Table 4:** Theme of impact of media on vaccine acceptance.

	**Caregivers’ Comments**
Botswana	• “We also get information on radios, on TVs.” “Sometimes the same media issues are so distorted that you may want to turn off your radios because there would be so much negative issues that would make one doubt the safety of vaccines.” • “There are also radio programs that address immunization services as well as other health topics. Besides radios some health information is usually in newspapers.”
DR	• “I heard on the television that they were saying that they were going to integrate it into the immunization program, and I am praying that God that they will start giving it, because they told me it can prevent ear infections and other things.” • “On television, there are lots of announcements that you should get your children vaccinated.” • “When there is a virus it is always in the news, and I have heard that you should bring your child to be vaccinated when they hit a certain age, but only when there is a virus.” • “I think that if there was more information on the television, internet or radio, it would be a lot better. I never have heard anything on the television, or internet, or radio that talks about vaccines, never.”
Greece	• “Truly, through the internet and printed words, they are trying to induce parents not to go along with specific vaccines.” • “The Media brainwash.” • “I’ve read it on google.”
	**Health Care Providers’ Comments**
Botswana	• “At least that information is going quite well, when the campaigns are going on in Botswana, they do a really good media propaganda through TV, radio, and newspapers, and the awareness is coming very well actually.” • “So in the same way, when we hear of stories that people have been vaccinated and it has turned out another way, you tend to lose faith on that vaccine or on vaccinations completely.” • “Yeah usually if something is said on radio or newspapers they will come in numbers.”
DR	• “You can get information from the internet, but this is sometimes limited.” • “What people can access is negative information associated with vaccines from other countries.” • “The Ministry of Public Health has implemented some televised programs, they even have a web site, they use mass media that the public has access to, mostly to encourage them.” • “The impact that the radio, TV and social networks may have is critical. Here at the hospital, we now see even low-income mothers have a cell phone.”
Greece	• “I mean they have been informed through the internet, and after all these years, still come back with the same question, which has not been altered, not even a little?” • “Then there is the internet, where parents enter and start to doubt…”I read that this vaccine caused these side effects and I decided not to do it to my child.”

DR, Dominican Republic

Across all three sites in both caregiver and provider focus groups, the theme of insufficient or ineffective communication about vaccines was prevalent, especially in Greece (Table 5). Caregivers were asked the question, “Overall, do you think there is enough information provided about the vaccines your children need?” while providers were asked, “Overall, do you think there is enough information provided about the vaccines you administer to children? In Botswana, providers indicated that increasing education through media with positive messages might be helpful. Caregivers suggested a number of methods to increase information availability, including increasing information on “Under 5” cards, increasing media presence such as billboards, and having more information sessions presented by health care providers. In the DR, more information was requested to increase caregivers’ knowledge and for clarification of confusing messages. In response to the question, “What else would be helpful to know about vaccines?,” caregivers requested more information through radio, television, and internet sources, whereas providers suggested increased information dissemination from the public health system. Providers also noted the distance that many people live from health care settings and the need to disseminate information through newspapers or television more frequently than just during outbreaks.

**Table 5:** Theme of insufficient communication.

	**Caregivers’ Comments**
Botswana	• “One-on-one education as opposed to, or even group education as opposed to, more TV or more radio or something.” • “We need workshops or at least group discussions like this particular one. It helps us learn new things and understand some of the things deeper.” • “Many a times these are people who would have had no opportunity to learn about these vaccine-preventable diseases. Usually in such cases, it becomes very hard to understand and for someone to even agree to have their children vaccinated because they are not sure of the nature of the disease.”
DR	• “I agree that there should be more information because, for example, I am in this discussion but there are things that are not clear about vaccines.” • “On television it only talks about dengue and cholera, and nothing more.”
Greece	• “There are pediatricians who support ‘yes, you do all the vaccines’ and there are some others who don’t support and that is where we get confused regarding what we should do.” • “But when the doctor’s profession, the doctors, their opinions differ, I mean some are in favor and some against, you can only imagine.“ • “In low society levels people don’t have the knowledge nor the education. He does it, I do it too.” • “No. I don’t know many things. There isn’t anybody to inform us…”
	**Health Care Providers’ Comments**
Botswana	• “If we don’t advertise these vaccines through the media, just waiting for people to come to the clinic and that is when you teach them, it is not sufficient.” • “It is only when there is an outbreak, outbreak or when there is a, what is the word to use, when there is a campaign, measles campaign that is when we tend to advertise more on the TV and the radio, but otherwise apart from that you will hardly hear of vaccines.” • “Yes, so that the can have at least a full course on the vaccines because there they are just to pass by, they don’t teach us in details about the vaccines we just learn on the vaccines when we are at the field.”
DR	• “Yes, of course, because if there was enough information, we would not have patients dying.” • “Unfortunately, there isn’t adequate information that can reach people from all income levels, because yes, the web is great, but how many computers will you find in La Ciénaga, or those marginal areas, or in faraway rural areas? It’s different with information you may get on the radio. The radio can give you the information, maybe not daily, but at least once a month. Have one day for an immunization campaign, because there are really no immunization campaigns.” • “I think that we still have to keep trying, with the television media, because there are communities where newspapers don’t arrive. And if they do, the morning paper gets there in the afternoon. I mean there are places where newspapers never reach, and they don’t even have TV.” • “There aren’t any television or radio audiovisual campaigns that consistently inform the residents, there really aren’t.”
Greece	• “It would be good for only experts to talk about vaccines, like infectious disease specialists and pediatricians. Other people may have the knowledge but they don’t have the experience.” • “That is the hard part, because parents are bombarded from all sides.” • “In the new health booklet there is analytic information on what each vaccine protects from. No one opens and reads them.” • “From the parents’ side, yes, there is lack of information.”

DR, Dominican Republic

In Greece, caregivers described the need for more information on vaccination and for better dissemination strategies. Many participants reported inconsistent messaging coming from health care providers often creating confusion among caregivers. Ineffective communication by the media, specifically on the internet, in the form of either unclear or too much information, was also cited. Caregivers suggested that they would appreciate more reliable, consistent information from media sources (such as television) as well as information circulating through the educational system. To minimize conflicting statements, they also expressed the desire for a single responsible body that would coordinate and communicate up-to-date and consistent information about vaccines.

### Domain: Attitudes and interactions with the health care system

In Botswana, participants expressed an overwhelming trust in the government and closely aligned the health care system and government as one unit. Interactions with health care workers were equally positive and negative, with reliance on health care providers for information yet concerns about providers’ knowledge, skills, or compassion. In contrast in the DR, there were few comments related to trust in the system or government. Similar to Botswana, participants reported experiences with health care workers that both supported and set up barriers to immunization. In Greece, participants reported significant barriers related to access to vaccination and a lack of public health infrastructure. This resulted in caregivers choosing to obtain vaccines from the pharmacy and pay for vaccine administration in the private sector rather than trying to navigate the public system. Representative quotes are highlighted in Table 6 and Table 7.

**Table 6:** Trust and interactions in the health care system: Caregivers’ comments.

	**Trust in the Health Care System Supports Acceptance**
Botswana	“Normally the government here won’t just have a vaccine that they haven’t tested or they researched about. They won’t even use something that would risk our babies’ lives.”
	**Interactions with Health Care Workers Support Acceptance**
DR	“They motivate us to bring [our children] to get vaccinated, and sometimes they bring the vaccines to us.”
Greece	“You take your child to the pediatrician and the pediatrician tells you that he has done all the vaccines until now, you are not stressed.”
	**Interactions with Health Care Workers are a Barrier to Acceptance**
Botswana	“At times, you even wonder if there is a commitment when the health workers go for a long break forgetting that some people were there since this morning.”
DR	“Also, there are nurses who do a bad job with shots.”
Greece	“It makes it incredibly difficult for us. Everyone is moving toward the private sector and the pediatricians, and buy them and many are not prescribed.”

DR, Dominican Republic

**Table 7:** Trust and interactions in the health care system: Health care providers’ comments.

	**Interactions with Health Care Workers Support Acceptance**
Botswana	“Just like they trust me and respect me with handling of their children as far as other diseases are concerned, they handle vaccines in a similar way, if they can trust me then yes I can vaccinate their child, they trust that I am doing the right things for their child…”
DR	“At the PAI, you know, the immunization program that operates at the public health level, they do a great job. Those people know what they are doing.”
Greece	“At the mother child health centers…there are some doctors contracted with the public health schemes where you can go right now.”
	**Interactions with Health Care Workers are a Barrier to Acceptance**
DR	“Well, in my case, there are some patients that complain about some of the vaccine posts, or rather about the attitude of the vaccinator in certain sites, i.e., ‘I don’t go to that one place because the person acts a certain way.’ “
Greece	There are millions of uninsured people, 3 million in a population of 10 million people is a big number, who cannot vaccinate their children.”

DR, Dominican Republic; PAI, Programa Ampliado de Inmunización

## Discussion

In this qualitative study, we explored the role of knowledge and communication on vaccine acceptance in countries with both resource limitations and rapidly changing immunization programs. While there is a growing body of literature investigating drivers of vaccine acceptance, few studies have focused on the impact of different methods of information dissemination on vaccination programs in middle-income countries with expanding immunization programs. Major findings from this work demonstrate that while both caregivers and immunization providers from a wide range of communities in Botswana, the DR, and Greece report high levels of support for vaccination because of knowledge of vaccines and vaccine-preventable diseases, a need still exists for increased access to accurate and reliable information about vaccines. Contrasts in responses between countries highlighted the importance of information sources including health care providers, medical literature, and the media in supporting vaccine-related knowledge. For example, while the media primarily supported positive vaccine beliefs and was viewed as a way to disseminate messages to encourage vaccination in the DR and Botswana, media messages were largely viewed as negative in Greece. There were also differences in the degree of trust in the public health system across sites. These differences highlight the influence of context on vaccine acceptance and emphasize the importance of region- or country-specific research to understand local drivers of vaccine acceptance.

While disseminating scientifically sound information about vaccines is critical, reliable information alone does not necessarily promote sufficient trust in vaccines to influence decision making about vaccines, especially among individuals with strongly held beliefs [9]. Recognizing which source of information is most relied upon by caregivers and providers and which communication method is most effective is essential when considering strategies to improve local vaccine acceptance. Provider recommendation is known to be a key predictor of vaccine acceptance [35,36]. As demonstrated in the theme of health care providers and medical literature as an information source, participants across all three sites relied on health care providers as their primary source of information, although many participants from Greece identified conflicting messages from providers as an issue, highlighting the importance of consistency. In contrast, in Botswana, health care providers were overwhelmingly recognized as the trusted, consistent information source for caregivers, especially when confronted with negative media messages.

Beyond direct communication with health care providers, there are multiple ways to disseminate information about vaccines. Participants’ comments about the use of multiple types of media highlight this idea. However, research establishing the effectiveness of different communication strategies on changing vaccination behavior is in its infancy [22,37]. Mass media campaigns are often used by public health systems to promote health messages and have been associated with improved child health outcomes [38]. This study focused on the role of media given the proliferation of social media outlets and rapid dissemination of both pro- and anti-vaccine messages. In Botswana and the DR, popular media as a tool for the dissemination of positive messages was perceived to be a potentially effective tool. Negative vaccine messages in the popular media were not viewed as reliable and, therefore, had little to no influence on attitudes and beliefs about vaccines. In contrast, in Greece, the media, specifically the internet, was more often described as disseminating negative messages that did not support vaccine acceptance. Additionally, such messages were often considered in vaccine decision-making. While the comments we elicited demonstrate the varied types of media available to participants in different regions, the theme of insufficient communication highlights that both caregivers and providers are eager for more substantial, accurate information from reliable sources, regardless of the medium through which it is communicated.

A lack of confidence in vaccines in the face of a knowledgeable public is known as the vaccine confidence gap and indicates that an additional mix of factors affects the public’s trust and subsequent vaccine acceptance. In fact, citizens’ trust in immunization programs and the health care system requires understanding the complex religious, socioeconomic, historical, and political contexts of different regions [39]. The multiple references to trust in health care workers and the health care system reflects this concept. In Botswana, our results showed a high level of trust in the government and heath care system as a provider of resources and information. In contrast, trust in the health care system and government was very low among Greek respondents, which may reflect the recent economic instability and loss of many government services for a large proportion of the population. Our results illustrate the complexity of factors influencing vaccine acceptance and also suggest potential targets to enhance the success of immunization programs.

### Possible limitations of findings

This is a qualitative study among a sample of caregivers and immunization providers. While we recruited from regions of each country and from both private and public health care settings, our population is a non-random sample of each country’s population. Recruitment strategies in each country were designed with local collaborators, but certain populations in each country may be underrepresented in our sample. In the DR and Botswana, we primarily recruited through clinics. There may be unique facilitators of and barriers to vaccination in other locations where health care services may be more dispersed or among families that do not interact with the health care system for preventive care. In Greece, we were able to capture a range of social classes in Athens as well as in the semi-urban area because of recruitment through the kindergarten networks.

We have identified dominant themes but qualitative research cannot quantitatively measure predictors of vaccine acceptance. Counts of dominant themes as presented are hypothesis-generating and can inform the development of survey instruments that can be used in future quantitative studies across a broader range of communities and health care delivery settings in each country for more generalizable data. Our focus group guide was based upon a conceptual model grounded in behavioral theories, but it is possible that we may have missed additional salient themes. However, we attempted to address this by adding themes that emerged during the coding process.

### Implications

Prior studies have indicated that knowledge about vaccination is important but not sufficient to change behavior [22]. In this study, participants communicated an understanding of the need for vaccination and had a significant understanding of vaccines and vaccine-preventable diseases; however, participants also expressed interest in receiving more information about vaccination. To address some of the challenges identified by participants related to knowledge and attitudes, the vaccine delivery system has the powerful ability to be an influential and cost-effective purveyor of vaccine information.

In a country such as Botswana where trust in government is high, this trust can be leveraged to bolster communication about vaccines. In the DR, such messages may be more effective if disseminated through an individual’s health care provider or social network, as study participants did not voice significant trust in the public health system. In Greece, participants emphasized the dependency on health care providers to help guide decision-making about vaccines yet also reported inconsistent information. To support effective communication and ensure consistency, providers can be targeted for vaccine education interventions. This may be an important mechanism to strengthen trust in health care systems and help close the vaccine confidence gap. Future quantitative work based on this study should focus on identifying characteristics of populations that correlate with vaccine acceptance and on identifying strategies to remedy the identified barriers.

Vaccine delivery and acceptance are complex issues affected by a variety of local and personal factors. These data reinforce the notion that while some facilitators and barriers to vaccination may be generalized, an understanding of local determinants is crucial to supporting effective implementation of immunization programs. For these reasons, working with local teams and developing materials that address local situations and concerns are imperative to successful implementation of new or maintenance of existing vaccine programs.

## Supporting information

S1 Appendix Tables present counts of the frequency with which each theme was discussed, categorized as either supporting vaccine acceptance, creating a barrier to vaccine acceptance, or without impact on vaccine acceptance. (DOCX)
