# Supplementary material for: The impact of access to immunization information on vaccine acceptance in three countries
Source: PLoS One. 2017 Aug 3;12(8):e0180759. doi: 10.1371/journal.pone.0180759 (PMC5542683; doi:10.1371/journal.pone.0180759)
Supplement: S1 Appendix — (DOCX) [file pone.0180759.s001.docx]

**S1 Appendix:** Tables present counts of the frequency with which each theme was discussed, categorized as either supporting vaccine acceptance, creating a barrier to vaccine acceptance, or without impact on vaccine acceptance.

**QUANTITATIVE RESULTS**

**Domain: Knowledge and Communication**

|  | **Dominican Republic** | **Greece** | **Botswana** |
| --- | --- | --- | --- |
| *Social norms (what other community members do)* |  | 43 |  |
| Supports acceptance | 25 | 45 | 36 |
| Barrier to acceptance | 46 | 62 | 4 |
| *Vaccine and vaccine-preventable disease knowledge* |  | 23 |  |
| Supports acceptance | 143 | 21 | 86 |
| Barrier to acceptance | 47 | 19 | 32 |
| *General communication about vaccination* |  | 54 |  |
| *Health care provider as information source (includes medical literature)* |  | 39 |  |
| Positive | 137 | 36 | 159 |
| Negative | 3 | 30 | 1 |
| No influence | 2 | 2 | 7 |
| *Community members (nonmedical) as information source* |  | 11 |  |
| Positive | 21 | 1 | 13 |
| Negative | 7 | 14 | 9 |
| No influence | 0 | 0 | 3 |
| *Media* |  | 24 |  |
| Positive message | 41 | 10 | 35 |
| Negative message | 9 | 34 | 14 |
| No influence | 9 | 7 | 37 |
| *Insufficient communication available* | 59 | 109 | 56 |

**Domain: Interactions with the Health Care System**

|  | **Dominican Republic** | **Greece** | **Botswana** |
| --- | --- | --- | --- |
| *Attitude toward health care system and government* |  | 18 |  |
| Trust | 15 | 38 | 80 |
| Distrust | 6 | 47 | 14 |
| *Interactions with health care workers* |  | 5 |  |
| Supports acceptance | 20 | 2 | 28 |
| Barrier to Acceptance | 25 | 4 | 31 |

**Domain: Attitudes and Beliefs**

|  | **Dominican Republic** | **Greece** | **Botswana** |
| --- | --- | --- | --- |
| *General Attitudes and Beliefs* |  | 23 |  |
| *Beliefs about vaccine safety* |  | 1 |  |
| Supports acceptance | 60 | 12 | 10 |
| Barrier to acceptance | 92 | 50 | 73 |
| *Beliefs about vaccine efficacy* |  | 1 |  |
| Supports acceptance | 84 | 41 | 60 |
| Barrier to acceptance | 6 | 12 | 14 |
| *Perceived risk of vaccine-preventable disease* |  | 5 |  |
| Supports acceptance | 44 | 27 | 39 |
| Barrier to acceptance | 15 | 29 | 2 |
| *Experience with vaccine-preventable disease* |  | 10 |  |
| Supports acceptance | 32 | 10 | 16 |
| Barrier to acceptance | 7 | 13 | 3 |
| *Religious and Spiritual Beliefs* |  | 0 |  |
| Supports acceptance | 3 | 0 | 2 |
| Decreases acceptance | 10 | 3 | 38 |
| *Experience and beliefs with alternatives to vaccination* |  | 12 |  |
| Supports acceptance |  | 6 |  |
| Barrier to acceptance |  | 26 |  |
| *Decision-Making Responsibilities/Concessions* |  | 59 |  |
| Supports vaccination |  | 6 |  |
| Barrier to vaccination |  | 6 |  |
| Inability to reach concession |  | 1 |  |

**Domain: Logistics of Vaccine Delivery**

|  | **Dominican Republic** | **Greece** | **Botswana** |
| --- | --- | --- | --- |
| *Cost* |  | 6 |  |
| Supports acceptance | 11 | 19 | 9 |
| Barrier to acceptance | 46 | 38 | 6 |
| *Time* |  | 1 |  |
| Supports acceptance | 24 | 3 | 9 |
| Barrier to acceptance | 16 | 6 | 32 |
| *Travel & Logistics* |  | 3 |  |
| Supports acceptance | 35 | 7 | 17 |
| Barrier to acceptance | 57 | 27 | 38 |
| *Supply* |  |  |  |
| Supports acceptance | 18 | 1 | 27 |
| Barrier to acceptance | 44 | 16 | 39 |
| *Literacy* |  |  |  |
| Supports acceptance | 3 | 0 | 3 |
| Barrier to acceptance | 1 | 0 | 8 |
| *Incentives* |  | 3 |  |
| Supports acceptance | 4 | 37 | 22 |
| Barrier to acceptance | 0 | 7 | 2 |

**FOCUS GROUP SCRIPT - CAREGIVERS**

Welcome. Thank you for attending today’s focus group. Everyone in this group cares for a child who either has received or can receive immunizations. We are hoping to get a discussion going about what you know about the vaccines your child receives, how you learn about vaccines and what you think about them. Every ones ideas are important so please give everyone an opportunity to speak. Also, everything is confidential and should not be discussed outside of this room. Since we will be taping this discussion, I ask that we use only first names today. Thank you.

ICEBREAKER- Before we start, let’s get to know each other a little bit. How many children do you have and what are their ages?

Let’s start by thinking about the vaccines your child receives. What do you think vaccines do for your child?

Prompts:

- Tell me more
- Let me see if I understand correctly what you are telling me

Now let’s talk about why you think people get their children vaccinated. I want you to each think of three or four reasons people get their children vaccinated. Think about the things that make it easier to get children vaccinated or things people may believe. This time we will start here (*point to one person*) and go around the room with each person giving me one reason they think people get their children vaccinated. I will write your ideas on this board and we will keep going around the room until everyone feels that the list is complete.

Prompts:

- Tell me more
- Let me see if I understand correctly what you are telling me

Now let’s talk about what why you think people do not get their children vaccinated. I want you to each think of three or four reasons people do not get their children vaccinated. Think about the things that make it more difficult to get children or vaccinated or things people may believe. This time we will start here (*point to one person*) and again, go around the room with each person giving me one reason they think people do not get their children vaccinated. Again I will write your ideas on this board and we will keep going around the room until everyone feels that the list is complete.

Prompts:

- Tell me more
- Let me see if I understand correctly what you are telling me

Now I am going to ask you about some reasons other people have talked about. What I want to hear from you is if you think these things are important or not and why they are or are not important. (*Discuss those domains not already discussed previously including:*

*Lack of information about recommended vaccines*

*Lack of information about diseases prevented by the vaccines*

*Have had or know someone who has had a disease prevented by vaccines*

*Other families in community get their children (or do not get their children) vaccinated*

*Clinic staff recommends vaccines*

*Fear that child will get sick if not vaccinated*

*Desire to protect others in community*

*Belief that it is better to let children develop natural immunity*

*Belief that my child is not at risk of getting some of the diseases prevented by vaccines*

*Belief that vaccines are not safe*

*Belief that vaccines cause the diseases against which they are meant to protect*

*Belief that vaccines cause other diseases*

*Belief that vaccines do not work*

*Belief that vaccines contain harmful ingredients*

*Difficulty getting to a clinic for vaccinations because of travel time or work schedule*

*Long waiting time at clinics*

Where do you get information about the vaccines your child needs?

Prompts:

- What do you think is the best way to find out about vaccines?
- Which sources do you trust the most for information about vaccines?
- Where do you usually go to get other kinds of health information?
- How do you use this information to make decisions about your child’s immunizations?

Sometimes there are stories in the news about vaccines. How do these news stories change what you think about vaccines?

Prompts:

- Has a story you heard in the news made you ask for (or look for) more information about a vaccine?

Overall, do you think there is enough information provided about the vaccines your children need?

Prompts:

- What else would be helpful to know about vaccines?
- How do you know when your child needs a vaccine?

How does the health care system in general make it easier or harder for you to get vaccines for your child?

Prompts:

- Do you feel like you can talk to the clinic staff about questions you might have about vaccines? Does it matter?
- Do you feel like the clinic staff listens to you? Does it matter?
- Does the clinic staff treat you with respect? Does it matter?
- Do you trust the immunization provider (your doctor/nurse)? Does it matter?

Are there other vaccines you would like to see offered at your health center / clinic?

**FOCUS GROUP SCRIPT- IMMUNIZATION PROVIDERS**

Welcome. Thank you for attending today’s focus group. Everyone in this group works in a health center or clinic and administers immunizations to children. We are hoping to get a discussion going about what you know about the vaccines you administer to children, how you learn about the vaccines and what you think about them. Everyone’s ideas are important so please give everyone an opportunity to speak. Also, everything is confidential and should not be discussed outside of this room. Since we will be taping this discussion, I ask that we use only first names today. Thank you.

Let’s start by thinking about what you know about the vaccines that are routinely recommended for children. What do you think vaccines do for the children that you see?

Now let’s talk about why you think people get their children vaccinated. I want you to each think of three or four reasons people get their children vaccinated. Think about the things that make it easier to get children vaccinated or things people may believe. We will start here (*point to one person*) and go around the room with each person giving me one reason they think people get their children vaccinated. I will write your ideas on this board and we will keep going around the room until everyone feels that the list is complete.

Prompts:

- Tell me more
- Let me see if I understand correctly what you are telling me

Now let’s talk about what why you think people do not get their children vaccinated. I want you to each think of three or four reasons people do not get their children vaccinated. Think about the things that make it more difficult to get children or vaccinated or things people may believe. This time we will start here (*point to one person*) and go around the room with each person giving me one reason they think people do not get their children vaccinated. Again I will write your ideas on this board and we will keep going around the room until everyone feels that the list is complete.

Prompts:

- Tell me more
- Let me see if I understand correctly what you are telling me

Now I am going to ask you about some reasons other people have talked about. What I want to hear from you is if you think these things are important or not and why they are or are not important. *Discuss those domains not already discussed previously including:*

*Lack of information about recommended vaccines*

*Lack of information about diseases prevented by the vaccines*

*Have had or know someone who has had a disease prevented by vaccines*

*Other families in community get their children (or do not get their children) vaccinated*

*Clinic staff recommends vaccines*

*Fear that child will get sick if not vaccinated*

*Desire to protect others in community*

*Belief that it is better to let children develop natural immunity*

*Belief that children are not at risk of getting some of the diseases prevented by vaccines*

*Belief that vaccines are not safe*

*Belief that vaccines cause the diseases against which they are meant to protect*

*Belief that vaccines cause other diseases*

*Belief that vaccines do not work*

*Belief that vaccines contain harmful ingredients*

*Difficulty getting to a clinic for vaccinations because of travel time or needing to take*

*time from work*

*Long waiting time at clinics*

Where do you get information about the vaccines you administer to children?

Prompts:

- What sources do you consider to be most accurate?
- What sources do you consider to be most trustworthy?

Sometimes there are stories in the news about vaccines. How do these news stories change what you think about vaccines?

Prompts:

- Has a story you heard in the news made you ask for (or look for) more information about a vaccine?
- Has a story you heard in the news made you think about not giving certain vaccines to children that you see?
- Have parents asked you about news stories they have heard about vaccines?

Overall, do you think there is enough information provided about the vaccines you administer to children?

Prompts:

- Do you feel that you are able to answer parents’ questions about vaccines?
- What else would you like to know about the vaccines that you administer?
- What do you think is the best way for parents to find out about vaccines?
- Do you feel like parents know when their child is due for a vaccine?
- Do you feel like there is enough advertisement about the immunization program? Does it matter?
- How do you respond when a family refuses a vaccine?

Does the health care system in general make it easier or harder for you to get vaccines for children?

Prompts:

- Do you feel like you have enough vaccines to give to all of the children who come to an immunization clinic? Have you ever had to turn a family away because there was not enough vaccine available?
- Do you feel like your health center has the right supplies to properly store vaccines?
- Do families treat you with respect? Does it matter?
- Do you feel respected by your supervisors? Does it matter?

Are there other vaccines you would like to see offered at your health center / clinic?

- How do you think the immunization program can best accommodate any new vaccines?
- How do you think that families will accept the addition of any new vaccines the immunization schedule?
